# MycoDB, a global database of plant response to mycorrhizal fungi

**DOI:** 10.1038/sdata.2016.28

**Published:** 2016-05-10

**Authors:** V. Bala Chaudhary, Megan A. Rúa, Anita Antoninka, James D. Bever, Jeffery Cannon, Ashley Craig, Jessica Duchicela, Alicia Frame, Monique Gardes, Catherine Gehring, Michelle Ha, Miranda Hart, Jacob Hopkins, Baoming Ji, Nancy Collins Johnson, Wittaya Kaonongbua, Justine Karst, Roger T. Koide, Louis J. Lamit, James Meadow, Brook G. Milligan, John C. Moore, Thomas H. Pendergast IV, Bridget Piculell, Blake Ramsby, Suzanne Simard, Shubha Shrestha, James Umbanhowar, Wolfgang Viechtbauer, Lawrence Walters, Gail W. T. Wilson, Peter C. Zee, Jason D. Hoeksema

**Affiliations:** 1 Department of Environmental Science and Studies, DePaul University, Chicago, Illinois 60614, USA; 2 National Institute for Mathematical and Biological Synthesis, University of Tennessee, Knoxville, Tennessee 37996-3410, USA; 3 Department of Biology, University of Mississippi, University, Mississippi 38677, USA; 4 School of Forestry, Northern Arizona University, Flagstaff, Arizona 86011, USA; 5 Department of Ecology and Evolutionary Biology, University of Kansas, Lawrence, Kansas 66045, USA; 6 Colorado Forest Restoration Institute, Colorado State University, Fort Collins, Colorado 80523-1472, USA; 7 Department of Biological Sciences, Northern Arizona University, Flagstaff, Arizona 86011, USA; 8 Department of Biology, Indiana University, Bloomington, Indiana 47405, USA; 9 Departamento de Ciencias de la Vida, Universidad de las Fuerzas Armadas—ESPE, Sangolquí 1715231B, Ecuador; 10 US Environmental Protection Agency, Office of Solid Waste and Emergency Response, Washington DC 20004, USA; 11 Université Toulouse 3 Paul Sabatier, CNRS, ENFA; UMR5174 EDB (Évolution & Diversité Biologique); F-31062 Toulouse, France; 12 Department of Biology, University of British Columbia Okanagan, Kelowna BC, Canada V1V1V7; 13 College of Forestry, Beijing Forestry University, Beijing 100083, China; 14 School of Earth Sciences and Environmental Sustainability, Northern Arizona University, Flagstaff Arizona 86011, USA; 15 Department of Microbiology, Faculty of Science, King Mongkut’s University of Technology Thonburi, Bangkok 10140, Thailand; 16 Department of Renewable Resources, University of Alberta, Edmonton, Canada T6G 2E3; 17 Department of Biology, Brigham Young University, Provo, Utah 84602, USA; 18 School of Forest Resources and Environmental Science, Michigan Technological University, Houghton, Michigan 49931-1295, USA; 19 Institute of Ecology and Evolution, University of Oregon, Eugene, Oregon 97403, USA; 20 Department of Land Resources and Environmental Sciences, Montana State University, Bozeman, Montana 59717, USA; 21 Department of Biology, New Mexico State University, Las Cruces, New Mexico 88003, USA; 22 Department of Ecosystem Science and Sustainability, and the Natural Resource Ecology Laboratory, Colorado State University, Fort Collins, Colorado 80523, USA; 23 Department of Plant Biology, University of Georgia, Athens, Georgia 30602, USA; 24 Department of Forest and Conservation Sciences, University of British Columbia, Vancouver, British Columbia, Canada V6T 1Z4; 25 Department of Biological Sciences, Winston Salem State University, Winston-Salem, North Carolina 27110, USA; 26 Department of Biology, University of North Carolina, Chapel Hill, North Carolina 27599, USA; 27 Department of Psychiatry and Neuropsychology, Maastricht University, 6200 MD Maastricht, The Netherlands; 28 Software Engineering, Enova International Inc., Chicago, Illinois 60604, USA; 29 Department of Natural Resource Ecology and Management, Oklahoma State University, Stillwater, Oklahoma 74078, USA; 30 Department of Biology, California State University—Northridge, Northridge, California 91330, USA

**Keywords:** Ecology, Phylogenetics, Fungal ecology, Arbuscular mycorrhiza, Fungal evolution

## Abstract

Plants form belowground associations with mycorrhizal fungi in one of the most common symbioses on Earth. However, few large-scale generalizations exist for the structure and function of mycorrhizal symbioses, as the nature of this relationship varies from mutualistic to parasitic and is largely context-dependent. We announce the public release of MycoDB, a database of 4,010 studies (from 438 unique publications) to aid in multi-factor meta-analyses elucidating the ecological and evolutionary context in which mycorrhizal fungi alter plant productivity. Over 10 years with nearly 80 collaborators, we compiled data on the response of plant biomass to mycorrhizal fungal inoculation, including meta-analysis metrics and 24 additional explanatory variables that describe the biotic and abiotic context of each study. We also include phylogenetic trees for all plants and fungi in the database. To our knowledge, MycoDB is the largest ecological meta-analysis database. We aim to share these data to highlight significant gaps in mycorrhizal research and encourage synthesis to explore the ecological and evolutionary generalities that govern mycorrhizal functioning in ecosystems.

## Background & Summary

Plant performance is largely a function of the plant-symbiotic microbiome^1^. As a result, ecosystem functions and the vital services humans derive from them (e.g., food and fiber production, carbon sequestration) are fundamentally dependent on the interactions plants have with symbionts. Although symbioses are common, our knowledge of their impact on ecosystem functions and services is relatively incomplete. Broad generalizations about the relationships between plants and their symbionts are limited due to the context-dependent nature of such symbioses, which exist along a continuum of possible outcomes, from mutualistic to parasitic. Examining the results of many experiments through meta-analysis allows for larger-scale generalizations than individual experiments can provide independently and can lead to synthesis-generated evidence^2^. Furthermore, including phylogenetic information in meta-analyses can account for correlated evolutionary relationships among taxa used in multiple studies. Understanding ecological outcomes of symbioses, and the environmental and evolutionary context contributing to such outcomes, is crucial to maintaining and restoring the ecosystem functions and services upon which humans depend.

Mycorrhizal fungi form an ancient symbiosis with most plants on Earth^3,4^. The host plant and associated fungi form a trading partnership where the fungi increase the effective absorptive capabilities of the plant, delivering nutrients and water in exchange for plant-derived photosynthates^5^. Since most plants associate with mycorrhizal fungi, the outcome of this symbiosis can influence ecosystem structure, function, and services mediated by plant productivity. Several empirical studies have individually demonstrated how host plant traits and identities, fungal partners, soil biotic and abiotic conditions, and experimental conditions can alter the structure and function of mycorrhizal symbioses^6–9^. The important role of mycorrhizal fungi in global change dynamics such as N deposition, climate change, and invasive species has also been documented^10–12^ as well as the key role that mycorrhizal fungi play in the restoration and conservation management of ecosystems^13–15^. Understanding the generality of how environmental context impacts the relationship of plants with their mycorrhizal symbionts should also affect how we manage terrestrial ecosystems.

Statistical methods to simultaneously examine multiple ecological and evolutionary factors in a meta-analysis framework have been recently developed^16–18^, facilitating computational analytic approaches to studying global patterns of mycorrhizal symbioses. Large datasets with multiple moderators are vital to such approaches because ecological meta-analyses based on small datasets can be vulnerable to spurious interpretations resulting from unbalanced data distributions, correlated moderators, and unavailable information regarding potentially important predictors. For example, a meta-analysis of 51 studies^19^ suggested the absence of synergy between mycorrhizal fungi and nitrogen-fixing bacteria likely resulted from an overrepresentation of annual and agricultural species in the meta-analysis database; by contrast, separate tests of late successional legumes demonstrated strong synergism^20^. Another meta-analysis^21^ suggested that the paradoxical result of declining plant growth response to mycorrhizal inoculation with nitrogen but not phosphorus fertilization was due to the overrepresentation of studies in the dataset with high soil phosphorus and the potential correlation among moderators. The problem of spurious interpretations within ecological meta-analyses can be reduced by simultaneous testing of multiple predictors, which is made possible by larger databases.

**Figure 1:**
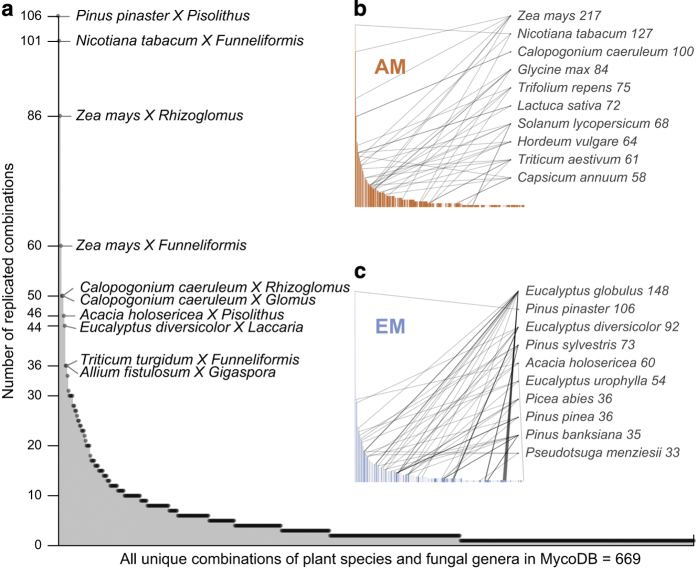
The distribution of unique plant species and fungal genus combinations contained in MycoDB. Larger figure (**a**) shows the overall distribution of unique plant fungal combinations (669 total) in the database with the most common combination being *Pinus pinaster* inoculated with *Pisolithus* (106 studies). Inset graphs separate AM (**b**) and EM (**c**) fungal studies and highlight the 10 most common plant species in each subset. Numbers next to plant names on insets indicate the quantity of studies in MycoDB that utilize that plant host. Lines next to plant names indicate the number of fungal genera used to inoculate a particular plant species across studies.

**Figure 2:**
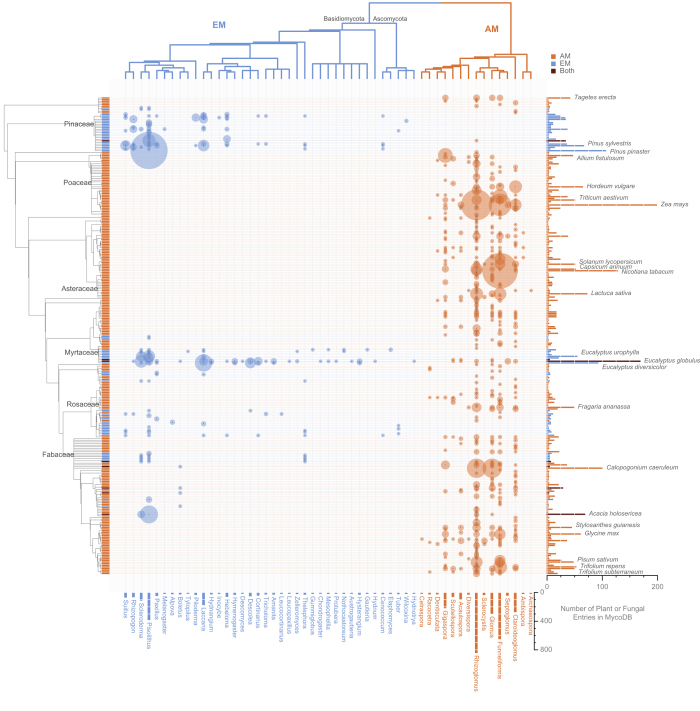
Heat map showing the frequency of studies in MycoDB according to unique plant host and fungal genus combinations and their phylogenetic relationships. Larger circles indicate a larger number of studies that exist in MycoDB for that unique plant species-fungal genus pair. The left side shows a composite phylogenetic tree for plant species, and a composite phylogenetic tree for mycorrhizal fungal genera is on the top of the graph. Different colors indicate plants grown in association with different mycorrhiza types (blue=EM fungi, red=AM fungi, black=either AM or EM).

Here we present MycoDB, a large database of mycorrhizal inoculation experiments, linked with plant and fungal phylogenies (Data Citation 1), to facilitate tests on the ecological and evolutionary contexts in which the addition of mycorrhizal fungi is beneficial or parasitic to plant hosts. MycoDB focuses on studies of two dominant types of mycorrhizal fungi, ectomycorrhizal (EM) fungi and arbuscular mycorrhizal (AM) fungi, because they predominate among published studies on mycorrhizal symbioses. MycoDB contains data on plant productivity response to mycorrhizal fungi from 4,010 studies (from 438 unique publications) and is organized in a hierarchical fashion such that a single publication can contain multiple discrete experiments and a single experiment can contain multiple studies. The ecological and evolutionary context of studies can be explored with 24 additional explanatory variables (e.g., plant functional group, inoculum complexity, plant or fungal origin; Table 1 (available online only) and Table 2) and mycorrhizal fungal and plant host phylogenetic trees (Figs 1 and 2). MycoDB can be used to model phylogenetic heritability of plant response to mycorrhizal fungi in host plant lineages, fungal lineages, and their interaction, as well as explore the relationship among explanatory variables and plant response to mycorrhizal fungi, while controlling for the influence of plant and fungal phylogenies.

## Methods

### Overview and literature searches

MycoDB (Data Citation 1) contains data from three main phases of data collection and validation. Phase I occurred in 2005, when we identified 1852 publications by conducting an initial literature search of the ISI Web of Science database using the key words mycorrhiz* and inocul* (on January 22, 2005). From this initial list, 134 publications were selected, in random order, as having met our inclusion criteria for meta-analysis such as reporting plant biomass response, use of a mycorrhizal addition treatment, and inclusion of a non-inoculated control (see ‘Criteria for inclusion’ below). More publications from the initial list of 1852 publications likely met our criteria, but were excluded from Phase I of database construction because of time constraints. Data from 49 additional publications on EM fungi were added from a previous meta-analysis^22^ to reduce dominance of the data by studies on AM symbioses. This process resulted in a total of 183 publications summarized in MycoDB during Phase I. In 2010, as part of an NCEAS (National Center for Ecological Analysis and Synthesis) Distributed Graduate Seminar conducted across nine institutions, we began Phase II of the data collection to dramatically increase the size of MycoDB with several targeted literature searches. On September 21, 2010, we conducted searches of the ISI Web of Science database using the following search terms: (1) (mycorrhiz* or ectomyc* or endomyc* or arbuscul* or vesicular*) and inocul* resulting in 4,013 papers; (2) search terms from (1) AND *restoration* or *rehabilitation* or *reclamation* or *revegetation* or *reforestation* resulting in 305 papers; (3) search terms from (1) AND *local adaptation* or *strain* or *isolate* or *genotype* or *ecotype* or geograph* resulting in 627 papers; (4) search terms from (1) AND *tissue P* or *tissue N* or *shoot P* or *shoot N* or *leaf P* or *leaf N* resulting in 793 papers; (5) search terms from (1) AND Gigaspor* or Acaulospor* or *Scutellospora* or *Archaeospora* resulting in 387 papers. Searches 2–5 were designed to enrich the database in studies relevant to restoration ecology, local adaptation, influences of nutrients, and AM fungi besides *Glomus*, because these topics were identified either as being of interest for planned focused meta-analyses (restoration, local adaptation) or as under-represented in the Phase I search results (influences of nutrients, AM fungi besides *Glomus*). The results from all five searches were collated, duplicates (as well as publications already included in MycoDB) were removed, and papers were selected that appeared in at least one of the four focused searches (2–5), resulting in a list of 1,768 publications. Again, from this larger list, 255 publications were selected, in random order, as having met our inclusion criteria for meta-analysis such as reporting plant biomass response, use of a mycorrhizal addition treatment, and inclusion of a non-inoculated control (see below ‘Criteria for inclusion’), and their data were added to MycoDB. After Phase II, MycoDB contained data from a total of 4,010 studies from 438 publications. Phase III of the creation of MycoDB consisted of extensive data validation and the creation of phylogenetic trees for all plant species and fungal genera in the database.

A subset of MycoDB was used to study how edaphic properties, plant functional groups, and microbial community complexity determine the outcome of mycorrhizal symbioses^21^. Different subsets of these data have been used to explore local adaptation among plants, mycorrhizal fungi, and soils (Rúa *et al.* in review) as well as how partner identity, colonization levels, and P fertilization impact plant host response to EM associations^22^.

### Criteria for inclusion

Prior to inclusion in MycoDB, publications were screened for meta-analysis appropriateness and for an experimental design that was amenable to our research questions. It was required that all studies compare results of a mycorrhizal inoculation treatment (or several treatments) to a non-inoculated control. In other words, studies must compare plant response for some addition of mycorrhizal fungi to no addition. The method of inoculation varies among studies in MycoDB and can include the addition of spores, roots, mycelia, pot culture, field soil, or any combination thereof. Studies that apply mycorrhizal fungi to all treatments, eliminate fungal presence (e.g., using fungicide application), or otherwise manipulate ecological factors to promote or suppress fungi were not included. We included studies with unsterilized background soil, many of which likely contained propagules of mycorrhizal fungi in all treatments, though this could not be confirmed because fungal colonization data was not consistently reported. If an experiment contained the manipulation of a factor in addition to mycorrhizal fungi (e.g., fertilizer treatment, soil amendment), the results are included in MycoDB as separate studies within the same experiment. All studies report mean plant biomass data as the response variable. Studies could report shoot biomass, total biomass, or root biomass and shoot biomass. Studies must report means, but were still included if measures of dispersion (e.g., standard error, standard deviation, or error bars on figures) were not given as was the case in 91% of studies. If sample size was not given, the associated parameter ‘n’ was coded as 1 for both inoculated treatments and non-inoculated controls, reducing the weight of the study relative to what it would be if the sample size was known. Data presented in tables were extracted directly, but data presented in figures were extracted using Engauge Digitizer software version 4.1^23^. Data on additional explanatory variables (e.g., plant family, plant functional group) were also extracted from the publication text when available or looked up using supplementary peer-reviewed resources. Both lab studies and field studies are included in MycoDB and coded separately using the variable LOCATION (Table 1 (available online only)). No limitation was placed on the duration of study for inclusion in MycoDB; in the case of studies that examined plant biomass over a time series, only data from the last sampling event was included. Data were then entered into MycoDB using a custom web-based data entry interface and database that matched inoculated treatments with non-inoculated controls^24^.

### Plant and fungal phylogenetic tree construction

We constructed plant and fungal phylogenetic trees for all the species in MycoDB using a composite phylogeny approach, which combines taxonomic and phylogenetic information into a single tree^16,25,26^. For plant phylogeny, we derived phylogenetic topology from existing ‘supertrees’ and assigned well-supported divergence times to all possible internal bifurcations (evolutionary divergence event) using TimeTree^27^ as a source of published divergence times. The remaining unknown branch lengths were rooted with known divergence dates^28^ and arbitrarily set and scaled to yield an ultrametric tree wherein all extant species were lined up at the present date. In cases where taxonomic nomenclature has changed since the creation of the original ‘supertrees’ or publication of papers used in the database, names were changed manually to reflect current consensus taxonomy. Pairwise shared branch-lengths were then used to calculate a variance-covariance matrix, which can be used in mixed multifactor meta-analyses.

For the fungal composite phylogeny, we manually reconstructed the evolutionary relationships among different genera based on known or commonly accepted taxonomy using information from previously published reports. Fungal taxonomy, particularly of AM fungi, has undergone major revisions during the duration of the compilation of MycoDB. We traced the evolution of fungal taxa into current consensus systematics^29^; however, because of ambiguity in species identification of AM fungi, we only included fungal taxonomic identification to the genus level. Even so, some taxa formerly named *Glomus* could not be placed definitively within current genera and were therefore left as *Glomus*. In the case of the AM fungal phylogenetic branch, the composite tree topologies between different genera of this clade were informed by taxonomic position of the type species of each genus (when possible) in relationship with the taxonomic position of the other type species of another genus^29–31^. For EM fungi, the position of each species in the phylogenetic tree follows the online version of Index Fungorum (www.indexfungorum.org) and recent taxonomic literature. The phylogenetic framework and tree topology were based on the 2007 AFTOL classification of Fungi^32^ and other recent efforts in fungal systematics^33,34^.

Construction and manipulation of composite phylogenies was conducted using R Statistical Software^35^ (version 3.0.2), the *ape* package in R^36^, Phylocom^37^, and Phylomatic^37^. The files contain the fungal and plant composite phylogenies in the Newick tree format. The FungalTree_version1.txt file (Data Citation 1) represents the evolutionary relationships among different fungal genera that exist in the MycoDB database. Similarly, the PlantTree_version1.txt file (Data Citation 1) represents the evolutionary relationships between different plant species that exist in this database, with each node of the plant’s composite tree labeled with corresponding higher taxonomic classification.

### Data attributes

Data in MycoDB are organized in a hierarchical manner as a single publication often contained data from multiple discrete experiments and studies (i.e., trials) on multiple plant hosts. As such, the 438 publications in MycoDB contain data for 4,010 studies (Data Citation 1). A study is defined as a comparison of average plant performance between plants that were inoculated with mycorrhizal fungi (AM or EM, never both) and plants that were not inoculated. Table 1 (available online only) contains detailed meta-data for all variables in MycoDB including descriptions of variables and levels. Figure 1 demonstrates the frequency and distribution of unique plant species and fungal genus combinations (669 total) contained in MycoDB. For example, the most frequently reported mycorrhizal combination was *Pinus pinaster* (maritime pine) inoculated with species from the EM fungal genus *Pisolithus* (106 studies). The two inlay graphs represent the most common plant species in MycoDB, separated by mycorrhizae type (AM vs EM). Lines to plant species indicate the number of fungal genera in association with each plant species. For example, the most common plant species in MycoDB are *Zea mays* (corn, 217 studies) and *Eucalyptus globulus* (blue gum, 148 studies), in association with AM fungi and EM fungi, respectively. Figure 2 is a heat map representing the frequency of studies according to unique plant host and fungal genus combinations and their phylogenetic relationships. For EM fungi, the most commonly represented plant-fungal combinations in MycoDB occur between plants within the Pinaceae growing in association with *Pisolithus* fungi. For AM fungi, hotspots occur within the Poaceae, Solanaceae, and Fabaceae grown in association with *Rhizoglomus* and *Funneliformis* fungi. As these plant families are important to forestry and agriculture, their prevalence in the literature makes sense, but the tropics and thus a large portion of plant and fungal biodiversity are underrepresented. Figure 2 suggests that empirical work thus far regarding the mycorrhizal symbiosis is not only limited with respect to the plant and fungal species examined, but also relatively poorly represented among phylogenetically diverse clades of plants and fungi.

Although this database represents the efforts of over 80 people distributed over 10 years, these data still only represent a fraction of the total available data on plant response to mycorrhizal fungi. The number of papers published each year that fit our search criteria has grown exponentially in the time since our initial search. The 351 plant species in our database represent a small proportion of the 450,000 total plant species on Earth^38^, the majority of which likely associate with mycorrhizal fungi. Moreover, as might be expected, the plant taxa represented are heavily biased toward species important for agriculture and forestry (e.g., corn, tobacco, pine, eucalyptus). Similarly, the fungal taxa that are best represented in the database are taxa that have been commercially marketed, such as the ectomycorrhizal fungus *Pisolithus tinctorius*^39^. Given this uneven representation of plant and fungal species and potential correlations among closely related plant and fungal species, it is important to analyze these data using phylogenetic mixed models even when testing environmental moderators of plant responsiveness to mycorrhizal fungal inoculation.

### Statistical considerations

MycoDB was prepared in anticipation of common technical problems (and statistical solutions thereof) encountered in meta-analyses. In particular, a difficulty in many ecological meta-analyses is the lack of independence of the estimates. Multiple estimates (‘studies’) extracted from the same publication may be more similar to each other than those arising from different publications due to similarities in experimental methods or context within the same publication. Multilevel meta-analytic models (with estimates nested within publications) can be used to account for such correlated data structures^18^. Similarly, multiple estimates may represent contrasts of different treatments that are compared against a common control condition, leading to statistical dependencies in the estimates due to reuse of information from the control condition^40^. Hence, identification of estimates that share a common control condition is of crucial importance (i.e., variable CTLTRTSETID in Table 1 (available online only)). Moreover, multiple studies may use the same species or different species that are phylogenetically related, and such taxonomic overrepresentation may limit the scale of inference of the meta-analysis. Inclusion of information on phylogenetic relations within a mixed-effects model can account for these correlations and allow tests of generality of results. This can be done through inclusion of categorical taxonomic (e.g., family, genus, species) variables as random effects^21^ or through an analysis that includes the full phylogeny^41,42^. Including phylogenetic information in meta-analyses can account for correlated evolutionary relationships as well as allow inference on the rates and constraints of evolution on the phenotypic character being considered^18^. Finally, the availability of phylogenies for both plant species and fungi offers the possibility of modeling potential coevolution and ecological interactions using appropriate mixed-effects models^43^.

As the largest database concerning this important symbiosis, MycoDB may prove particularly useful for the development of meta-analysis educational curriculum or statistics tutorials using ecological data. In a classroom setting, for example, the database could be used in demonstrations of single predictor meta-analyses, which could then be followed up with comparison of multiple moderator meta-analyses to demonstrate the consequences of correlated predictors. Subsets of the data could support investigation of the advantages of larger datasets in overcoming problems of correlated predictors, thereby promoting exploration of context-dependency in future meta-analyses.

## Data Records

### Data record 1

The database file in csv format, titled ‘MycoDB_version1.csv’ (February 5, 2016 version), was uploaded to the Dryad Digital Repository (Data Citation 1). Detailed meta-data for each column is located in Table 1 (available online only) of this Data Descriptor article. Taxonomic information on plant species and fungal genera contained in the database are included in Supplementary File 1 of this article.

### Data record 2

The phylogenetic tree of mycorrhizal fungal genera present in Data record 1, in txt format and titled ‘FungalTree_version1.txt’ (February 5, 2016 version), was uploaded to the Dryad Digital Repository (Data Citation 1). The file contains the fungal genera composite phylogeny in the Newick tree format.

### Data record 3

The phylogenetic tree of plant hosts present in Data record 1, in txt format and titled ‘PlantTree_version1.txt’ (February 5, 2016 version), was uploaded to the Dryad Digital Repository (Data Citation 1). The file contains the plant composite phylogeny in the Newick tree format.

## Technical Validation

We devised several layers of methods to ensure the quality of the data in MycoDB. First, random sampling of publications that resulted from our initial searches was conducted to reduce bias in which data were included in the database. Second, on the front end, data collection was conducted using a web-based custom data entry system with organized fields and drop down menus to reduce data entry error^24^. This approach also allowed data collection to be conducted simultaneously and remotely by multiple users. After front-end data entry by users, database administrators conducted back-end database content management to validate data integrity by examining distributions and outliers as well as iteratively hand-checking random subsets of papers for accuracy. We used the United States Department of Agriculture PLANTS Database^44^ and The Plant List version 1.1^45^ to verify and update scientific names and life histories for each species included in MycoDB. Database administrators hand checked outliers and returned to original papers when data were missing, as well as added moderator variables and edited moderator levels to facilitate specific meta-analyses. Finally, for the local adaptation study subset of MycoDB, all entries were compared with the original paper and corrected when necessary. The data validation methods used to create MycoDB satisfy all data-related compliance criteria designed to promote methodological quality in ecological meta-analyses^46^.

## Usage Notes

MycoDB is deposited in the Dryad Digital Repository at http://dx.doi.org/10.5061/dryad.723m1 and publicly available under the CC0 public domain dedication, given proper scholarly citation of the version used and this data descriptor. We recommend that, prior to publication, users validate data subsets against original publications.

## Additional information

**How to cite this article:** Chaudhary, V. B. *et al.* MycoDB, a global database of plant response to mycorrhizal fungi. *Sci. Data* 3:160028 doi: 10.1038/sdata.2016.28 (2016).

## Supplementary Material



Supplementary File 1

## Figures and Tables

**Table 1:** Summary of meta-data for each column in MycoDB including variable name, description, the type and range of data for each column, and the number of levels in each variable

**Variable**	**Description**	**Variable Type (range)**	**Levels (#studies/level)**
EXPERIMENTID	A numerical identifier for unique experiments. Publications can contain multiple experiments, which can contain multiple studies. Multiple studies may have the same EXPERIMENTID value.	Discrete (5 to 1460)	917
CTLTRTSETID	A numerical identifier for studies that use the same control mean value to calculate effect sizes. Multiple studies may have the same CTLTRTSETID value.	Discrete (78 to 15852)	2134
NONCTLTRTSETID	A unique numerical identifier for individual studies, i.e., observations of plant response to mycorrhizal inoculation.	Discrete (75 to 15854)	4010
LASTNAME1	Last name of the first author of the paper.	Categorical	334
LASTNAME2	Last name of the second author of the paper.	Categorical	316
PAPERYEAR	Year the paper was published.	Discrete (1976 to 2010)	34
JOURNALNAME	Name of journal in which the article was published.	Categorical	117
PAPERTITLE	Title of the publication.	Categorical	438
PAPERDATASOURCENAME	The database construction Phase in which a paper was included in MycodB.	Categorical	2004 Search (1698)Sept 2010 Main Search (2312)
EFFECTSIZE1	Effect size of fungal inoculation on plant biomass calculated as a log response ratio:$\ln\left(\frac{trt\_mass}{ctrl\_mass}\right)$Negative values indicate a parasitic plant-fungal relationship; values close to 0 indicate a commensalistic plant-fungal relationship; positive values indicate a mutualistic plant-fungal relationship.	Continuous (−4.68 to 7.38)	NA
ESTVAR1	Estimated within-study variance calculated as:$\sigma^{2}=\frac{1}{ctrl\_reps}+\frac{1}{trt\_reps}$	Continuous (0.01 to 2.0)	NA
ESTVAR3	Estimated within-study variance calculated according to Equation 1 from Hedges et al. (1999):$\sigma^{2}=\frac{trt\_sd^{2}}{trt\_reps\times trt\_mass^{2}}+\frac{ctrl\_sd^{2}}{ctrl\_reps\times ctrl\_mass^{2}}$	Continuous (0.000112 to 2.29)	NA
ctrl_mass	Average mass of plants from the non-inoculated control. Value refers to whole plant biomass if given. If not, value is shoot biomass. Units are in grams.	Continuous	NA
ctrl_reps	Number of replicates used for the non-inoculated control. This value was set as ‘1’ if the publication did not provide data on replication.	Continuous	NA
ctrl_sd	Standard deviation (s.d.) for average mass of plants from the non-inoculated control. s.d. was calculated from SE and other measures of dispersion. If none was given, this value was set to NA	Continuous	NA
trt_mass	Average mass of plants from the inoculated treatment. Value refers to whole plant biomass if given. If not, value is shoot biomass.	Continuous	NA
trt_reps	Number of replicates used for the inoculated treatment. This value was coded as ‘1’ if the publication did not provide data on replication	Continuous	NA
trt_sd	Standard deviation (s.d.) for average mass of plants from the inoculated treatment. s.d. was calculated from SE and other measures of dispersion. If none was given, this value was set to NA	Continuous	NA
PlantFamily	Family name of host plant	Categorical	72; see Supplementary File 1 for complete list
PlantSpecies	Plant host genus and specific epithet combined into a single variable (e.g., zea_mays)	Categorical	351; see Supplementary File 1 for complete list
FungalGenus	Fungal genus name according to phylogenetic structure as of July 2013.	Categorical	54; see Supplementary File 1 for complete list
PLANTLIFEHISTORY	Life history strategy of the plant host.	Categorical	annual_biennial (1331)perennial (2649)unknown (30)
FUNGROUP	Functional group category of host plant in study. Photosynthetic pathway, nitrogen fixing capabilities, and whether the plant is herbaceous (i.e., forb) or woody are summarized.	Categorical	C3 grass (297)C4 grass (328)Nfixforb (740)Nfixwood (426)nonNforb (853)nonNwood (1366)
NONMYCOCONTROL	Indicates whether additional measures were taken in a study to experimentally control for the effects of non-mycorrhizal soil microbes. Microbial wash indicates that either inoculum or experimental background soil was filtered in an attempt to remove mycorrhizal fungal propagules and then the filtrate was added to the non-inoculated control. Other indicates a different method was utilized to experimentally control for non-mycorrhizal microbes.	Categorical	microbial wash (756)none (2871)other (383)
NONMYCOCONTROL2	A consolidated version of NONMYCOCONTROL. Studies are coded as to whether non-mycorrhizal microbes were added or not.	Categorical	microbes_added (1139)mics_not_added (2871)
FERTP	Variable to indicate whether phosphorus fertilizer was added to both inoculated and control treatments.	Categorical	Pno (2151)Pyes (1859)
FERTN	Variable to indicate whether nitrogen fertilizer was added to both inoculated and control treatments.	Categorical	Nno (1699)Nyes (2311)
INOC.COMPLEXITY	Indicates whether multiple species or a single species of mycorrhizal fungi were added to inoculated treatments. For Whole, whole soil inoculum was added as inoculum, which likely contained multiple species of fungi, but it cannot be confirmed.	Categorical	Multi (369)Single (3499)Whole (142)
STERILIZED	Indicates whether the background soil was sterilized or not.	Categorical	STERno (1041)STERyes (2969)
MYCORRHIZAETYPE	Study examined the addition of either arbuscular mycorrhizal (AM) fungi or ectomycorrhizal (EM) fungi.	Categorical	AM (2984)EM (1026)
LOCATION	Describes the experimental setting of each study. Lab indicates any controlled environmental setting such as greenhouse or growth chamber.	Categorical	field (291)lab (3719)
DOMESTICATED	Describes the degree of agricultural domestication of plant host. Domesticated indicates plants bred selectively as crops or for ornamental purposes, forage crops are planted in pastures and available in a seed mix, and wild indicates no artificial selective breeding. Studies on EM fungi are listed as NA as they were not coded for domestication.	Categorical	DOMESTICATED (1912)FORAGECROP (321)WILD (751)NA (1026)
Rua2016	Indicates studies that were used in the Ruá et al. 2016 meta-analysis to explore local adaptation in AM fungi.	Categorical	NO (3683)YES (327)
Loc_Ad_appropriate	Indicates studies that have a known location of origin for at least two of the three experimental components (i.e., plants, fungi, soil) and could be assigned an LA_Code (see below).	Categorical	NO (2607)YES (1403)
LA_Code	Degree of sympatry or allopatry among plant host, fungal symbiont, and background soil for each study. NA indicates studies that were not coded for local adaptation and include all studies on EM fungi.	Categorical	See Table 2 for description of levels; NA (2609)
Plant_Lat	Latitude in decimal degrees of the origin location of the plant host.	Continuous (−35.3 to 69.0)	NA
Plant_Long	Longitude in decimal degrees of the origin location of the plant host.	Continuous (−157.8 to 157.7)	NA
Fung_Lat	Latitude in decimal degrees of the origin location of the fungal symbiont.	Continuous (−45.9 to 69.57)	NA
Fung_Long	Longitude in decimal degrees of the origin location of the fungal symbiont.	Continuous (−123.3 to 174.5)	NA
Soil_Lat	Latitude in decimal degrees of the origin location of the background soil.	Continuous (−40.6 to 63.4)	NA
Soil_Long	Longitude in decimal degrees of the origin location of the background soil.	Continuous (−123.2 to 158.0)	NA
AM_single_genus	Denotes studies where plants were inoculated with AM fungi from a single known genus, and therefore can be used in phylogenetic analyses.	Categorical	YES (2398)NO (1612)
EM_single_genus	Denotes studies where plants were inoculated with EM fungi from a single known genus, and therefore can be used in phylogenetic analyses.	Categorical	YES (1003)NO (3007)

**Table 2:** Description of LA_Codes such that components of studies—plants, fungi, and soil—share the same number if they originate from the same known location.

**LA_Code**	**Plant**	**Fungi**	**Soil**	**#studies**
A	1	1	1	96
B	1	1	2	8
C	1	2	1	57
D	2	1	1	14
E	1	2	3	104
F	1	1	X	64
G	1	2	X	77
H	1	1	Z	30
I	1	2	Z	140
J	1	X	1	11
K	1	X	2	63
L	X	1	1	96
M	X	1	2	299
N	Z	1	1	77
O	Z	1	2	265
Unknown locations are indicated by ‘X’. Artificial soils (e.g., peat moss) or non-wild plant varieties (e.g., cultivar or hybrid variety) are indicated by ‘Z’. For example, the database contains 299 studies, coded as ‘M’, where the source of the plant is unknown and the soil and AM fungi came from different locations.				
