# Supplementary material for: MycoDB, a global database of plant response to mycorrhizal fungi
Source: Sci Data. 2016 May 10;3:160028. doi: 10.1038/sdata.2016.28 (PMC4862322; doi:10.1038/sdata.2016.28)
Supplement: Supplementary File 1 [file sdata201628-s2.doc]

**Plant families in MycoDB**

Altingiaceae

Amaranthaceae

Amaryllidaceae

Anacardiaceae

Annonaceae

Apiaceae

Apocynaceae

Araceae

Araliaceae

Araucariaceae

Arecaceae

Asteraceae

Balsaminaceae

Berberidaceae

Betulaceae

Bignoniaceae

Boraginaceae

Bromeliaceae

Caryophyllaceae

Chenopodiaceae

Clusiaceae

Convolvulaceae

Coriariaceae

Cucurbitaceae

Cunoniaceae

Cupressaceae

Cyperaceae

Dipterocarpaceae

Elaeagnaceae

Erythroxylaceae

Euphorbiaceae

Fabaceae

Fagaceae

Geraniaceae

Juglandaceae

Lamiaceae

Lauraceae

Liliaceae

Linaceae

Magnoliaceae

Malvaceae

Melastomataceae

Meliaceae

Moraceae

Musaceae

Myrsinaceae

Myrtaceae

Nothofagaceae

Oleaceae

Orobanchaceae

Papaveraceae

Pedaliaceae

Pinaceae

Plantaginaceae

Poaceae

Polygonaceae

Primulaceae

Proteaceae

Ranunculaceae

Rhamnaceae

Rosaceae

Rubiaceae

Rutaceae

Salicaceae

Sapindaceae

Solanaceae

Thymelaeaceae

Ulmaceae

Urticaceae

Verbenaceae

Vitaceae

Zygophyllaceae

**Plant species in MycoDB**

*Acacia abyssinica*

*Acacia acuminata*

*Acacia ampliceps*

*Acacia auriculiformis*

*Acacia eriopoda*

*Acacia holosericea*

*Acacia monticola*

*Acacia nilotica*

*Acacia platycarpa*

*Acacia sclerosperma*

*Acacia seyal*

*Acacia sieberiana*

*Acacia* sp

*Acer platanoides*

*Acer pseudoplatanus*

*Adansonia digitata*

*Afzelia africana*

*Afzelia bella*

*Agrostis capillaris*

*Agrostis scabra*

*Agrostis stolonifera*

*Albizia lebbeck*

*Alchornea glandulosa*

*Allium cepa*

*Allium fistulosum*

*Allium porrum*

*Alnus acuminata*

*Alnus cordata*

*Alnus incana*

*Alnus tenuifolia*

*Anacardium occidentale*

*Anadenanthera peregrina*

*Ananas comosus*

*Andropogon gerardii*

*Anethum graveolens*

*Annona cacans*

*Anthonotha macrophyllum*

*Anthyllis cytisoides*

*Aphania senegalensis*

*Apium graveolens*

*Aquilaria filaria*

*Arachis hypogaea*

*Araucaria angustifolia*

*Aspalathus linearis*

*Asparagus officinalis*

*Aspidosperma parvifolium*

*Astragalus sinicus*

*Atriplex nummularia*

*Azadirachta indica*

*Balanites aegyptiaca*

*Berkheya coddii*

*Betula lenta*

*Bidens asymmetrica*

*Bidens sandvicensis*

*Bouteloua eriopoda*

*Bouteloua gracilis*

*Brachypodium pinnatum*

*Bromus inermis*

*Cabrelea canjerana*

*Caesalpinia ferrea*

*Caesalpinia peltophoroides*

*Cajanus cajan*

*Calamagrostis epigejos*

*Calliandra calothyrsus*

*Callistephus chinensis*

*Callitris rhomboidea*

*Calopogonium caeruleum*

*Canavalia ensiformis*

*Capsicum annuum*

*Carduus pycnocephalus*

*Castanopsis fissa*

*Catharanthus roseus*

*Cecropia pachystachya*

*Cedrela fissilis*

*Ceiba speciosa*

*Centrolobium tomentosum*

*Ceratopetalum apetalum*

*Chloris gayana*

*Chrysanthemum cinerariaefolium*

*Chrysanthemum morifolium*

*Cicer arietinum*

*Citrus aurantium*

*Citrus limonia*

*Citrus reshni*

*Citrus reticulata*

*Cladium jamaicense*

*Clusia minor*

*Clusia multiflora*

*Coffea arabica*

*Colophospermum mopane*

*Colubrina oppositifolia*

*Conyza bilbaoana*

*Copaifera langsdorffii*

*Cordia trichotoma*

*Cordyla pinnata*

*Coriandrum sativum*

*Coriaria nepalensis*

*Corymbia gummifera*

*Croton floribundus*

*Cryptosepalum tetraphyllum*

*Cucumis sativus*

*Cupressus atlantica*

*Cyamopsis tetragonoloba*

*Cyclamen persicum*

*Cymbopogon martinii*

*Cytharexyllum myrianthum*

*Dalbergia sissoo*

*Daucus carota*

*Dendrocalamus asper*

*Dendrocalamus strictus*

*Desmanthus illinoensis*

*Desmodium paniculatum*

*Desmoncus orthacanthos*

*Dialium guineensis*

*Dianthus caryophyllus*

*Dicorynia guianensis*

*Dodonaea triquetra*

*Dorycnium pentaphyllum*

*Dyera polyphylla*

*Eleusine coracana*

*Elsholtzia splendens*

*Elymus canadensis*

*Elymus elymoides*

*Eperua falcata*

*Eragrostis tef*

*Erythrina berteriana*

*Erythroxylum coca*

*Eucalyptus diversicolor*

*Eucalyptus europhylla*

*Eucalyptus globulus*

*Eucalyptus grandis*

*Eucalyptus hybrid*

*Eucalyptus loxophleba*

*Eucalyptus miniata*

*Eucalyptus pellita*

*Eucalyptus pilularis*

*Eucalyptus tetrodonta*

*Eucalyptus urophylla*

*Euphorbia pulcherrima*

*Fagus sylvatica*

*Faidherbia albida*

*Festuca arundinacea*

*Festuca rubra*

*Fragaria ananassa*

*Fraxinus excelsior*

*Gliricidia sepium*

*Glycine max*

*Gnaphalium californicum*

*Gossypium arboreum*

*Gossypium hirsutum*

*Guizotia abyssinica*

*Hakea preissii*

*Helianthus annuus*

*Hevea brasiliensis*

*Hippophae tibetana*

*Holcus lanatus*

*Homolepsis aturensis*

*Hopea helferi*

*Hopea odorata*

*Hordeum vulgare*

*Hymenaea courbaril*

*Impatiens balsamina*

*Ipomoea batatas*

*Ipomoea carnea*

*Juglans nigra*

*Juniperus oxycedrus*

*Kummerowia striata*

*Lactuca sativa*

*Lactuca serriola*

*Landolphia heudelottii*

*Lavandula spica*

*Lens culinaris*

*Lens esculenta*

*Leptospermum polygalifolium*

*Lespedeza bicolor*

*Leucaena leucocephala*

*Leymus arenarius*

*Linum usitatissimum*

*Liquidambar styraciflua*

*Litchi chinensis*

*Lithraea molleoides*

*Lolium perenne*

*Lolium rigidum*

*Lotus corniculatus*

*Luehea grandiflora*

*Lygeum spartum*

*Macaranga denticulata*

*Macherium stipitatum*

*Magnolia ovata*

*Manihot esculenta*

*Marlierea tomentosa*

*Medicago arborea*

*Medicago sativa*

*Medicago truncatula*

*Miscanthus sinensis*

*Morus alba*

*Musa aab*

*Musa acuminata*

*Myroxylon peruiferum*

*Myrsine umbellata*

*Myrtus communis*

*Nicotiana tabacum*

*Nothofagus dombeyi*

*Ocimum basilicum*

*Olea europaea*

*Origanum vulgare*

*Ormosia arborea*

*Oryza sativa*

*Osteomeles anthyllidifolia*

*Ostryopsis davidiana*

*Otholobium hirtum*

*Panax ginseng*

*Panicum amarum*

*Panicum miliaceum*

*Panicum virgatum*

*Papaver rhoeas*

*Paramacrolobium coeruleum*

*Parkia biglobosa*

*Paspalum conjugatum*

*Pelargonium peltatum*

*Persea americana*

*Petroselinum crispum*

*Petunia hybrida*

*Phaseolus vulgaris*

*Phleum pratense*

*Picea abies*

*Picea glauca*

*Picea mariana*

*Pinus banksiana*

*Pinus clausa*

*Pinus contorta*

*Pinus cornata*

*Pinus densiflora*

*Pinus elliottii*

*Pinus halepensis*

*Pinus jeffreyi*

*Pinus lambertiana*

*Pinus pinaster*

*Pinus pinea*

*Pinus ponderosa*

*Pinus radiata*

*Pinus resinosa*

*Pinus rigida*

*Pinus strobus*

*Pinus sylvestris*

*Pinus taeda*

*Pinus virginiana*

*Pistacia atlantica*

*Pistacia eurycarpa*

*Pistacia lentiscus*

*Pistacia terebinthus*

*Pistacia vera*

*Pisum sativum*

*Plantago lanceolata*

*Platyciamus regenellii*

*Platypodium elegans*

*Plectranthus amboinicus*

*Plectranthus barbatus*

*Podophyllum peltatum*

*Populus trichocarpa*

*Prosopis juliflora*

*Prunella vulgaris*

*Prunus avium*

*Prunus cerasifera*

*Prunus domestica*

*Prunus maritima*

*Prunus persica*

*Pseudotsuga menziesii*

*Psidium cattleianum*

*Pterocarpus officinalis*

*Pulsatilla patens*

*Pulsatilla pratensis*

*Quercus alba*

*Quercus faginea*

*Quercus ilex*

*Quercus petraea*

*Quercus robur*

*Quercus rubra*

*Quercus stellata*

*Quercus velutina*

*Retama sphaerocarpa*

*Rhamnus lycioides*

*Rhinanthus minor*

*Rhynchelytrum repens*

*Rosa multiflora*

*Rubus idaeus*

*Rumex acetosa*

*Saba senegalensis*

*Saccharum officinarum*

*Salix dasyclados*

*Salix fragilis*

*Salsola kali*

*Sapindus saponaria*

*Sauropus androgynu*

*Schinus terebinthifolius*

*Schizachyrium scoparium*

*Sclerocarya birrea*

*Senna macranthera*

*Senna reticulata*

*Senna siamea*

*Senna spectabilis*

*Sesamum indicum*

*Sesbania javanica*

*Sesbania sericea*

*Sesbania* sp

*Shorea pinanga*

*Shorea seminis*

*Sibbaldia procumbens*

*Solanum granulosoleprosum*

*Solanum lycopersicum*

*Solanum tuberosum*

*Solidago sempervirens*

*Sorghum bicolor*

*Sorghum sudanese*

*Spartium junceum*

*Stylosanthes guianesis*

*Syngonium podophyllum*

*Tabebuia impetiginosa*

*Tabebuia reseoalba*

*Tabebuia serratifolia*

*Tagetes erecta*

*Tamarindus indica*

*Tectona grandis*

*Tibouchina granulosa*

*Trachyspermum ammi*

*Trema micrantha*

*Trifolium alexandrium*

*Trifolium pratense*

*Trifolium repens*

*Trifolium subterraneum*

*Trigonella foenumgraecum*

*Triticum aestivum*

*Triticum durum*

*Triticum turgidum*

*Uapaca somon*

*Uniola paniculata*

*Veronica chamaedrys*

*Vicia cracca*

*Vicia faba*

*Vigna luteola*

*Vigna parkeri*

*Vigna radiata*

*Vigna unguiculata*

*Vitis berlandieri*

*Vitis riparia*

*Vitis rupestris*

*Vitis vinifera*

*Vulpia microstachys*

*Zea mays*

*Zinnia elegans*

*Ziziphus mauritiana*

**Fungal genera in MycoDB**

AM

*Acaulospora*

*Ambispora*

*Archaeospora*

*Cetraspora*

*Claroideoglomus*

*Dentiscutata*

*Diversispora*

*Funneliformis*

*Gigaspora*

*Glomus*

*Racocetra*

*Rhizoglomus*

*Sclerocystis*

*Scutellospora*

*Septoglomus*

EM

*Alpova*

*Amanita*

*Austrogautieria*

*Boletus*

*Cenococcum*

*Chondrogaster*

*Cortinarius*

*Descolea*

*Descomyces*

*Elaphomyces*

*Gautieria*

*Gummiglobus*

*Hebeloma*

*Hydnangium*

*Hydnotrya*

*Hydnum*

*Hymenogaster*

*Hysterangium*

*Inocybe*

*Laccaria*

*Leucocortinarius*

*Leucopaxillus*

*Melanogaster*

*Mesophellia*

*Nothocastoreum*

*Paxillus*

*Piloderma*

*Pisolithus*

*Protubera*

*Rhizopogon*

*Scleroderma*

*Suillus*

*Thelephora*

*Tricholoma*

*Tuber*

*Tylopilus*

*Wilcoxina*

*Zelleromyces*
